# Situational analysis of antibiotic prescriptions in Kenyan neonatal units for antimicrobial stewardship: a retrospective longitudinal study

**DOI:** 10.1016/j.eclinm.2025.103156

**Published:** 2025-03-26

**Authors:** Jalemba Aluvaala, Timothy Tuti, Muthoni Ogola, Cherry Lim, Sean Cavany, Mike English, The Clinical Information Network Author Group, Dolphine Mochache, Florence Murila, Wairimu Kimani, Duncan Chabi, Lilian Naibei, Juma Vitalis, Amilia Ngoda, Geoffrey Habil Shikanda, Nyumbile Bonface, Roselyn Malangachi, Ijusa Midecha, Eileen Muhavi, Samuel Soita, Christine Manyasi, Catherine Mutinda, Zanuba Mohammed, Rukia Aden, Rebecca Toroitich, Joyce Mbogho, Dion Nzoki, Joseph Ng’ang’a, Celia Kariuki, Cecilia Mutiso, Elizabeth Jowi, Josephine Aritho, Beatrice Njambi, Benjamin Wambua, Esther Mwangi, Charles Nzioki, Penina Musyoka, Zainabu Kioni, Miriam Munyalo, Esther Muthiani, Carol Ntii, Esther Njeri, Agnes Mithamo, Lucy Kinyua, Faith Kimotho, Magdalene Kuria, Alice Oguda, Mary Akoth, Christine Marete, Loise Mwangi, Mukami Becky, Penina Mwangi, Nancy Mburu, Juliet Gachoki, Rachel Inginia, Paul Njanwe, Mwende Mutunga, Celestine Muteshi, Ann Chebet, Emma Namulala, Yuvane Maiyo, Salome Muyale, Susan Wanjala, Grace Ochieng, Catherine Murianki, Lydia Thuranira, Virginiah Njoki, Margaret Waweru, Faith Mumo, Felistus Makokha, Maureen Natembea, Francis Soita, Josephine Ojigo, Maureen Muchela, Don Ogollah, Joyce Oketch, Assenath Okeyo, Rashid Musa, Beth Maina, Maureen Muriithi, Bashir Denkwo, Orina Nyakina, Faith Njeru, Judith Onsongo, Lucy Lyanda, Mwangi Wagura, Catherine Githaiga, Consolata Kinyua, Linda Ombito, Alice Nkirote, Elizabeth Kibaru, Caroline Limo, Benjamin Tanui, Patricia Muiruri, Bernadette Lusweti, Patrick Mburugu, Sylvia Mwathi, Maureen Njoroge, Marion Kiguoya, Jane Ndege, Peter Muigai

**Affiliations:** a Health Services and Implementation Cluster, KEMRI-Wellcome Trust Research Programme, P. O Box 43640 – 00100, Nairobi, Kenya; b Department of Paediatrics and Child Health, University of Nairobi, P. O. Box 19676-00202, Kenyatta National Hospital Nairobi, Nairobi, Kenya; c Centre for Global Health Research, Centre for Tropical Medicine and Global Health, Nuffield Department of Medicine, University of Oxford, Old Road Campus, OX3 7LG, Oxford, UK

**Keywords:** Neonatal, Antibiotics, Antibiotic use: antimicrobial stewardship

## Abstract

**Background:**

High antibiotic use in neonatal units may drive antimicrobial resistance and cause harm including mortality. We used data from 22 Kenyan neonatal units to (1) describe the proportion with antibiotic prescriptions at admission; (2) assess the predictors of non-first line antibiotic prescription; (3) estimate antibiotic use, and (4) explore postadmission antibiotic switching.

**Methods:**

Retrospective longitudinal study from 1st September 2020 to 31st October 2023. Antibiotics were classified as first line (penicillin plus gentamicin only), third generation cephalosporins (ceftazidime or ceftriaxone) or others. The proportion of antibiotic prescriptions were computed, and a multilevel logistic regression model used to analyse predictors of non-first line prescription. Antibiotic use was quantified by days of therapy (DOT) and length of therapy (LOT).

**Findings:**

Most neonates–62.6% (51,883/82,834)- received at least one antibiotic prescription at admission. Overall, first line antibiotics constituted 86% (44,636/51,883) but third generation cephalosporin use reached 100% in two facilities temporarily. The odds of non-first line prescription was greatest for outborn neonates (Odds ratio 2.27, 95% CI 2.12–2.43) while the estimated antibiotic consumption was 418 (389–500) per 1000 patient days by LOT and 744 (691–869) by DOT. From exploratory data post admission switching was most commonly to third generation cephalosporins.

**Interpretation:**

There is a high use of antibiotics potentially related to severity of illness at admission. Adherence to national guidelines for first line antibiotics is however generally high. Estimation of neonatal antibiotic prescription patterns and use over time and place is feasible and will be important in assessing the effectiveness of antimicrobial stewardship in Kenya and elsewhere in reducing antimicrobial resistance.

**Funding:**

This work was funded by the Wellcome Trust.

**Research in context**

**Evidence before this study**

We searched the Medline data base through PubMed for papers published from inception through to June 2024. The key words and search strategy used was (“neonate” AND “antibiotic” AND (“use” OR “consumption” OR “prescription”). Medical Subject Headlines (MeSH) were applied to the search terms where available. No language restriction was applied. We selected articles that included antibiotic consumption or use as defined by the WHO and reported a unit of measurement statistic.

Nine articles were included with five from low- and middle-income settings. These available data are few and with variation in metrics of antibiotic use plus lack of microbiology laboratory support. The Defined Daily Dose (DDD) is used in adults but is inappropriate in children due to weight-based dosing in the latter. Two use metrics that are applicable to children are the days of therapy (DOT) and length of therapy (LOT) based on number of antibiotics and days on antibiotics.

**Added value of this study**

Our study utilized routine data from 22 neonatal units and included more than 80, 000 admissions. Antibiotic use was analysed across time and place using proportions, DOT and LOT. These are critical for understanding variation in antibiotic use within and between hospitals over time as part of antibiotic surveillance and stewardship. In addition, we examined predictors of non-first line prescription at admission, identifying outborn babies–a heterogenous population–that are not covered in clinical practice guidelines as most likely to receive non-first line treatment. We also explored post admission antibiotic switching showing that a small proportion of sick newborns are subsequently prescribed third generation cephalosporins.

**Implications of all the available evidence**

This work shows that it is feasible to use routine data to implement surveillance of antibiotic use at scale in low resource settings using the neonatal unit as an exemplar. Such surveillance is a core component of antimicrobial stewardship and the fight against antimicrobial resistance.

## Introduction

Small and/or sick neonates are often prescribed antibiotics at admission to neonatal units following presentation with either risk factors for or signs and symptoms of possible severe infection.^1^^,^^2^ Given the large global burden of neonatal infection,^3^ the result is high antibiotic consumption. In settings with minimal routine microbiology diagnostic laboratory capacity like Kenya,^4^^,^^5^ high antibiotic consumption (including drugs like third generation cephalosporins), coupled with absence of antimicrobial stewardship may drive antimicrobial resistance.^2^^,^^6^^,^^7^ Moreover, vulnerable neonates may be exposed to potential harm including increased risk of invasive fungal infections, necrotizing enterocolitis, hearing loss and mortality.^6^

Available data on antibiotic use in neonatal units demonstrate variation in metrics of antibiotic use precluding direct comparison (Supplementary Table S1). The most reported antibiotic use metric was prevalence of antibiotic prescription This ranged from 70% by Murless-Collins et al. in four African countries^5^ to 23% by Spencer et al. in USA.^8^ The variation in antibiotic use metrics reflects a lack of consensus on their use in the neonatal population with a recent review providing a synthesis of available metrics with their pros and cons.^9^ The Defined Daily Dose (DDD) is the most common antibiotic use metric but is inappropriate in paediatrics due to weight based dosing. Newer metrics utilise days of antibiotics per 1000 patient days or length of stay (Days of Therapy (DOT) and Length of therapy (LOT)).^9^ These are appropriate for neonates and are reported in four out of the nine studies (Supplementary Table S1).^10^, ^11^, ^12^, ^13^

Antimicrobial stewardship (AMS) programmes provide a mechanism, with use metrics, for addressing antibiotic overuse in neonatal units to mitigate the attendant adverse outcomes.^1^^,^^14^ One core element of AMS is the availability of up-to-date, evidence based infection treatment guidelines which define appropriate and inappropriate antimicrobial use.^14^ Collection of antibiotic use data in conjunction with guideline derived measures of whether use is appropriate at facility level are then key components of a situational analysis in preparation for AMS programmes.^14^ In light of the few reports of antibiotic use data from low and middle income countries (LMIC) the aim was to quantify antibiotic use in 22 neonatal units in Kenya to identify opportunities for AMS interventions in a setting with agreed national guidelines that can help define inappropriate use. The objectives were to: (1) describe the proportion of neonates with antibiotic prescriptions (first line and others) over time and place linked to the distribution of key symptoms, signs and diagnoses at admission; (2) to assess the association of symptoms and signs with non-first line antibiotic prescription at admission; (3) to estimate consumption using the novel metrics Days of Therapy (DOT) and Length of therapy (LOT); and (4) to explore antibiotic switching post admission, which may indicate e.g. clinicians’ assessments that initial treatment is inadequate, particularly in settings where antibiotic choice is typically not informed by routine diagnostic microbiology.

## Methods

### Study design and setting

This was a retrospective longitudinal study that included 22 hospitals from the neonatal component of the Clinical Information Network (CIN) in Kenya.^15^ The CIN is a network of hospitals linked by a collaboration between, Kenya Medical Research Institute-Wellcome Trust Research Programme (KWTRP), the University of Nairobi, the Kenya Paediatric Research Consortium (KEPRECON), hospitals with their respective county governments and the national Ministry of Health. CIN aims to foster collaboration amongst its members with an aim of improving quality of inpatient neonatal and general paediatric care.^15^

The participating neonatal units offer essential intermediate level inpatient care for small and sick neonates apart from one facility which is a tertiary unit that provides intensive care.^16^^,^^17^ The tertiary unit offers routine microbiology laboratory services including blood cultures. Two of the other units were supported with some blood culture testing as part of a surveillance study on neonatal bacteraemia (November 2020 to November 2022).

### Study participants

All neonates admitted to the participating neonatal units from 1st September 2020 to 31st October 2023 and who stayed in the unit for up to 59 days (young infancy period) were eligible for inclusion. Exclusion criteria were age >28 days at admission, length of stay >59 days and missing treatment sheet (treatment sheets are the in-patient medical records on which clinicians prescribe).

### Variables

Antibiotics prescribed at admission were classified as “first line” i.e., crystalline penicillin and gentamicin as per the national guidelines,^18^ third generation cephalosporins or “others”. Antibiotic consumption at admission was quantified using Days of Therapy (DOT) and Length of therapy (LOT) defined in the section on statistical analysis.^9^ Antibiotic switch was defined as a prescription of a different antibiotic at least a day after the initial prescription at admission. If discharged without data on postadmission prescription it was assumed that the sole prescription was the admission one.

### Data sources and management

A key element of CIN is the collection of routine inpatient data in a process that has been described in detail previously.^17^^,^^19^ Data from all patients admitted to these units are entered into a Research Electronic Data Capture (REDCap©) based electronic data system at the point of exit by dedicated data clerks based in the facilities. The primary source documents are standardised clinical forms that are completed by the facility clinicians as part of routine patient care. The data entry process is supported by data quality assurance procedures at the point of entry and centrally at KWTRP.^19^ This includes quarterly double data entry on a sample of records to assess for concordance. Data are entered on all variables (full data set) or a minimum set of variables (minimum data set). The minimum data set are entered for (1) admissions during long holiday breaks, (2) admissions when the data clerk was on leave and (3) on a random selection of records in hospitals where the workload is very high.^19^ The postadmission antibiotic prescription data were entered non-systematically and to a different extent across the hospitals. This occurred where data clerks entered these additional data at variance with the standard operating procedure which only required prescriptions at admission.

### Statistical analysis

For descriptive statistics, categorical data were summarised as proportions and continuous data as medians (Interquartile range: IQR) if they were not normally distributed. Continuous variables were assessed for normality using histograms to assess whether the graph was relatively bell-shaped and symmetric around the mean. Antibiotic prescriptions at admission are tabulated as overall proportions and plotted as monthly proportions over the study period.

A multilevel logistic regression model considering clustering at hospital level was used to analyse the effect on prescription of non-first line antibiotic prescription at admission of: i) time (the study period) (ii) duration of hospital membership in CIN–N (number of months before September 2020, iii) membership of the Newborn Essential Solutions and Technologies programme (NEST360; an international collaboration implementing a package of care that includes affordable technologies, training and locally-owned data to deliver high quality small and sick newborn care),^5^ (iv) severity of illness (estimated probability of inpatient mortality using the score for essential neonatal symptoms and signs, SENSS^20^), v)Apgar score at 5 min, vi) oxygen saturation at admission (vii) birth outside the index hospital and, (viii) temperature at admission.

Antibiotic use was quantified by days of therapy (DOT) and length of therapy (LOT). DOT per day is defined as the number of days with ≥1 antibiotic divided by total patient days in hospital (facility-level) or individual patient length of stay (patient-level) where 3 drugs for one patient in one day equates to 3DOT.^9^^,^^21^ LOT per day is the number of days with ≥1 antibiotic divided by patient days (facility-level) or average length of stay (patient-level) but does not account for multiple drugs as is the case with DOT.^9^ Due to the limited availability of data on documentation of actual antibiotic administration computation of DOT and LOT to was restricted to *prescription at admission* and for both measures the prescribed number of days as the numerator (*prescribed DOT and LOT*) used. Post admission antibiotic switching was explored by using a Sankey plot.

### Reporting

The reporting of this work follows the Strengthening the Reporting of Observational Studies in Epidemiology (STROBE) statement for cohort studies.

### Patient and public involvement

There was no patient or public involvement in the design, conduct, or reporting of this work.

### Ethics

Ethical approval for collection of the CIN de-identified data is provided by the Kenya Medical Research Institute (KEMRI) Scientific and Ethics Review Unit (protocol no. SERU 3459). Individual patient consent was therefore not sought.

### Role of funding source

The funders had no role in study design; in the collection, analysis, and interpretation of data; in the writing of the report; and in the decision to submit the paper for publication.

## Results

### Study population

**Fig. 1:**
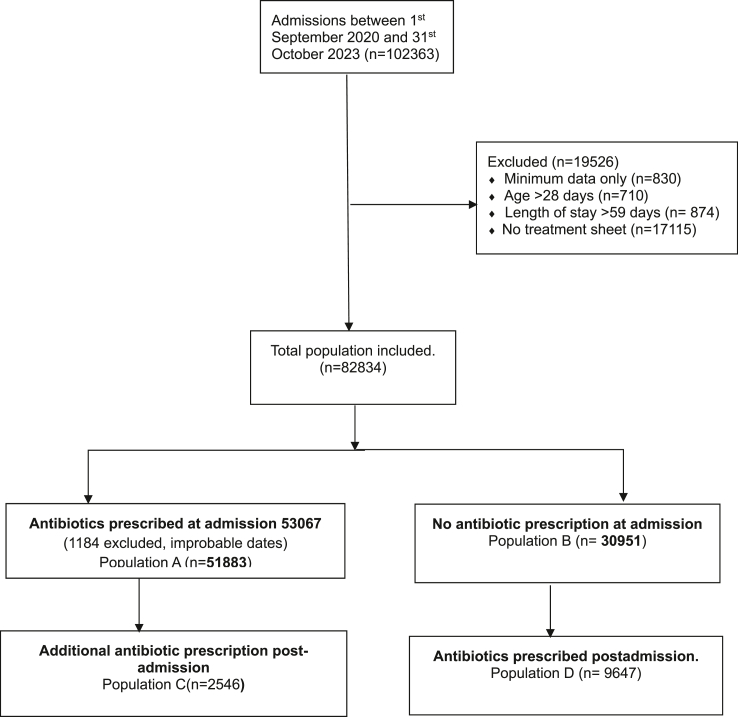
**Patient flow diagram**.

Twenty-two hospitals were included with a total of 102,360 admissions over the study period (Fig. 1) of which 101,533 had full data entered and were eligible for inclusion (n = 830 records with only minimum data that doesn't include treatments prescribed were excluded). There was variation in the number of neonates admitted across the hospitals during the study period ranging from 1676 to 38,637 (Supplementary Table S2). Further exclusions were age >28 days (n = 710), length of stay >59 days (n = 874), no treatment sheet (n = 17,115, treatment sheets are the in-patient medical records on which clinicians prescribe) and erroneous/improbable dates (1184) for a final sample of 82,834 (Fig. 1 and Table 1). Those excluded for improbable dates also had at least one other exclusion criteria and are therefore accounted for within the total 19,256 excluded (Fig. 1). Those excluded due to missing treatment sheets had less severe illness based on a lower score for essential neonatal symptoms and signs (SENSS) of 3.2 (IQR: 2.8–7.1) compared to 7.1 (IQR: 3–15.5) in the neonates included. Most of the neonates included- 51,883/82,834 (62.63%) had an antibiotic prescription at admission (Fig. 1 population A) while an additional 9647/82,834 (11.6%) who did not receive antibiotics on admission started treatment post-admission.

**Table 1:** Characteristics of the neonates admitted for inpatient care included in the study.

Indicator^b^	Treatment sheet present^a^ (n = 82,834)				Treatment sheet missing n= (17,115)	
	Antibiotics prescribed at admission (n = 51,883)^a^		Antibiotics not prescribed at admission (n = 30,951)^a^			
	Statistic^c^	Missing data	Statistic^c^	Missing data	Statistic^c^	Missing data
**Male (n, %)**	29,519 (56.9%)	146 (0.28%)	17,006 (54.94%)	90 (0.29%)	9444 (55.18%)	52 (0.3%)
**Birth weight**^d^, ^e^	2.77 (IQR: 1.95–3.24)	686 (1.36%)	2.6 (IQR: 1.8–3.2)	563 (1.82%)	2.958 (IQR: 2.3–3.4)	255 (1.49%)
ELBW	1581 (3.05%)	686 (1.32%)	971 (3.14%)	563 (1.82%)	328 (1.92%)	255 (1.49%)
VLBW	4440 (8.56%)		3044 (9.83%)		618 (3.61%)	
LBW	14,171 (27.31%)		9407 (30.39%)		4006 (23.41%)	
NBW	29,664 (57.17%)		15,488 (50.04%)		10,510 (61.41%)	
Macrosomia	1341 (2.58%)		1478 (4.78%)		1398 (8.17%)	
**Gestational age**^f^	38 (IQR: 34–39)	5507 (10.61%)	38 (IQR: 33–39)	3265 (10.55%)	38 (IQR: 36–40)	1813 (10.59%)
**Median length of stay (days)**	5 (IQR: 2–9)	0 (0%)	5 (IQR: 2–11)	0 (0%)	3 (IQR: 1–5)	0 (0%)
**Apgar 5 min < 7**	11,316 (21.81%)	4403 (8.49%)	5727 (18.5%)	2567 (8.29%)	1862 (10.88%)	1253 (7.32%)
**Tachypnoea**^g^, ^h^	16,243 (31.31%)	9297 (17.92%)	7289 (23.55%)	5629 (18.19%)	2864 (16.73%)	3933 (22.98%)
**Temperature**^i^						
Hyperthermia	5436 (10.48%)	8087 (15.59%)	1972 (6.37%)	4729 (15.28%)	732 (4.28%);	3076 (17.97%)
Normal	20,230 (38.99%)		13,323 (43.05%)		8243 (48.16%)	
Cold stress	11,044 (21.29%);		6600 (21.32%)		3534 (20.65%)	
Moderate hypothermia	6975 (13.44%)		4278 (13.82%)		1492 (8.72%)	
Severe hypothermia	111 (0.21%)		49 (0.16%)		38 (0.22%)	
**SpO**_**2**_**< 90%**^g^, ^j^	8998 (17.34%)	8245 (15.89%)	3826 (12.36%)	5313 (17.17%)	1337 (7.81%)	4352 (25.43%)
**SENSS score**^k^	7.1 (IQR: 3–15.5)	0 (0%)	5.4 (IQR: 3–13.4)	0 (0%)	3.2 (IQR: 2.8–7.1)	0 (0%)
Difficulty Feeding^g^	13,507 (26.03%)	2876 (5.54%)	6097 (19.7%)	2304 (7.44%)	1849 (10.8%)	1594 (9.31%)
Indrawing^g^	5012 (9.66%)	3224 (6.21%)	2076 (6.71%)	2757 (8.91%)	599 (3.5%)	1849 (10.8%)
Central Cyanosis^g^	3184 (6.14%)	2131 (4.11%)	1374 (4.44%)	1712 (5.53%)	470 (2.75%)	1508 (8.81%)
Floppy^g^	22,229 (42.84%)	5787 (11.15%)	11,006 (35.56%)	4840 (15.64%)	3543 (20.7%)	2285 (13.35%)
Convulsions^g^	3184 (6.14%)	2131 (4.11%)	1374 (4.44%)	1712 (5.53%)	234 (1.37%)	1489 (8.7%)
**Mortality**	8632 (16.64%)	37 (0.07%)	4589 (14.83%)	21 (0.07%)	1733 (10.13%)	41 (0.24%)

a Whether antibiotics have been prescribed at admission.

b Maternal indicators of PROM (Premature rupture of membranes), sepsis and fever had missingness rate of 91.01%, 95.05% and 49.26% respectively and were excluded.

c Continuous variable summaries given as median (Interquartile range). Discrete variable summaries given as proportions.

d Birth weight provided in kilograms.

e ELBW (Extremely Low Birth Weight): 1000 gms & Below; VLBW (Very LBW): 1001–1499 gms; LBW: 1500–2499 gms; NBW (Normal Birth Weight): 2500–4000 gms; Macrosomia: >4000 gms.

f Gestational age provided in weeks.

g Signs & Symptoms. 0: Absent, 1: Present.

h Fast breathing defined as respiratory rate >59 breaths/minute.

i In degrees Celsius, Hyperthermia: >37.5 °C, Normal Temperature: >36.5–37.5 °C; Cold stress: 36.0–36.4 °C; Moderate hypothermia: 32.0–35.9 °C; Severe hypothermia: <32.0 °C.

j SpO_2_: Oxygen saturation by pulse oximetry.

k SENSS (Score of Essential Newborn Symptoms and Signs) score provided as a mortality risk probability (derived from sex, birthweight, presence or absence of difficulty feeding, convulsions, indrawing, central cyanosis and floppy/inability to suck).

The median birthweight was 2.75 kg (IQR 1.9–3.2). All the admissions included had at least one sign of possible neonatal sepsis documented at admission (Table 1). Case fatality rate was 16%, 14% and 10% in those with antibiotic prescription at admission, no antibiotic prescription at admission, and no treatment sheets respectively.

**Fig. 2:**
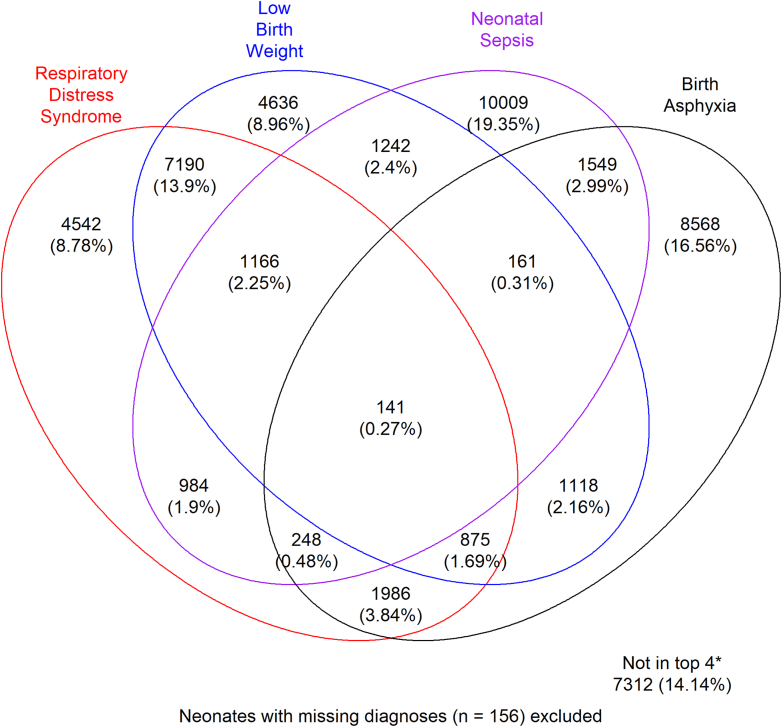
**Top four diagnoses for neonates with antibiotic prescriptions**. The areas of overlap are restricted to the top four diagnoses and depict instances where two or more of these were documented for a group of patients e.g. 1166 had low birth weight, respiratory distress syndrome, and neonatal sepsis. Where there is no overlap shows those who had only one of the top four diagnoses. Patients in either of these two categories (overlap versus no overlap) may also have another diagnosis in the “others” category.

In these settings with limited diagnostic capacity some patients had more than one diagnosis at admission and diagnoses were therefore counted as disease episodes (Fig. 2). Four diagnoses accounted for most (86%) antibiotic prescriptions i.e., preterm/low birth weight, respiratory distress syndrome, birth asphyxia and neonatal sepsis with a considerable number of patients having more than one of these diagnoses (Fig. 1). Low birth weight and respiratory distress syndrome were the most common at 17% each (Supplementary Table S3) followed by neonatal sepsis and birth asphyxia (15% each).

The other diagnoses included meconium aspiration, jaundice, meningitis, and congenital anomalies.

### Trends in antibiotics prescriptions at admission

Antibiotics prescribed at admission were classified as first line (penicillin *plus* gentamicin only), third generation cephalosporins only (ceftazidime or ceftriaxone) or others (penicillin, gentamicin, amikacin, ampicillin prescribed singly or in combination). The antibiotic prescription data included those with dates (for which the distinction between admission and postadmission could be made) and those without dates (Supplementary Table S4). Considering all these, other antibiotics included cephalosporins (1st, 2nd and 4th generation), penicillin's (amoxicillin, amoxicillin-clavulanate, flucloxacillin, cloxacillin, piperacillin-tazobactam), nitroimidazoles (metronidazole), sulphonamides (co-trimoxazole), quinolones (ciprofloxacin), lincosamides (clindamycin), glycopeptides (vancomycin), glycylcyclines (tigecycline). These were rare (<2% for each category) and were prescribed in both the tertiary and other facilities except for tigecycline used in only one case in the tertiary hospital.

**Fig. 3:**
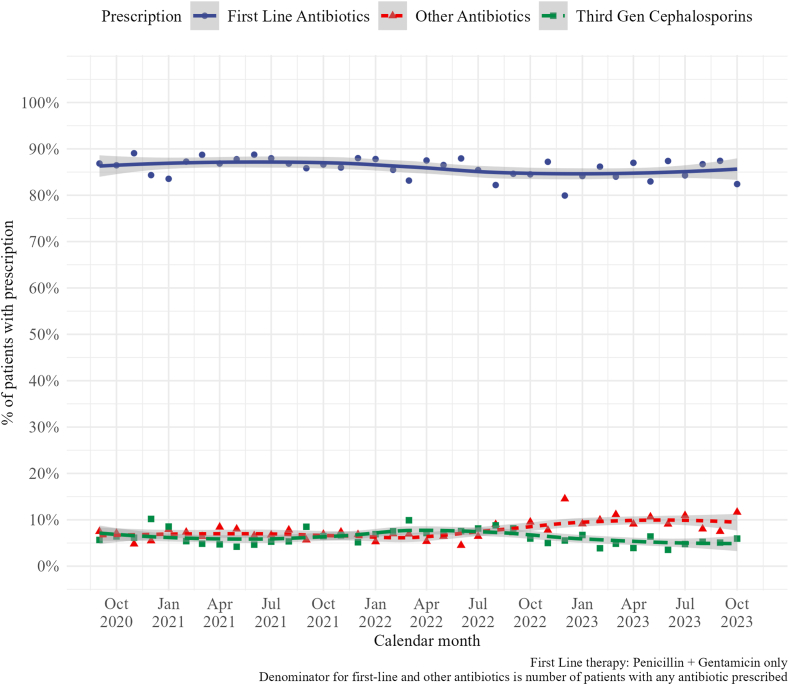
**Trend of antibiotic prescriptions at admission from September 2020 to October 2023**.

Over the study period across the whole network, the proportion of first line antibiotic prescriptions at admission (Fig. 1, population A) was 86% (44,636/51,883) with little change observed over time (Fig. 3). Results from a time series analysis using Generative Additive Models (GAMS) with a random slope for each hospital and smoothing terms for time, month of the year, and year in analysis, and an AR (1) (i.e., autocorrelation at 1 time step) show that the changes in first line antibiotic prescribing at admission are not statistically significant (IRR: 1.0058, 95% CI 0.9972–1.0144, p-value: 0.181) as shown in Supplementary Figure S3.

Minimal change was observed too for third generation cephalosporins (ceftriaxone or ceftazidime) and the “other” prescriptions at admission (Fig. 3) although a change from October 2022 with a small increase and decrease for “other” and “third generation cephalosporins respectively was noted. The non-overlapping confidence intervals after October 2022 suggests a difference in the proportion of “other” versus third generation cephalosporins prescribed.

**Fig. 4:**
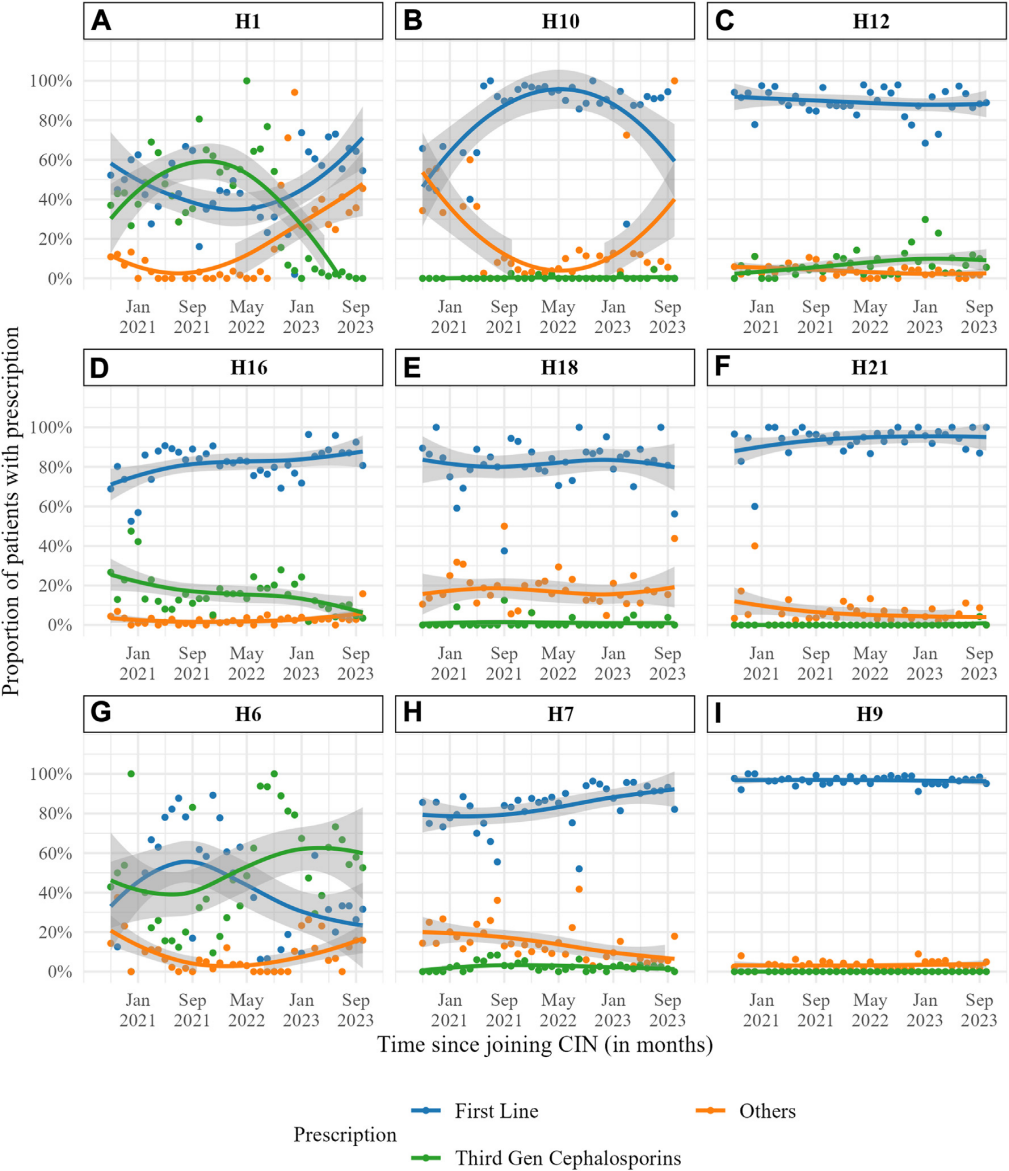
**Variation in antibiotic prescription patterns at admission in nine selected hospitals over time.** Individual dots represent the monthly proportion and the bold lines the trend line by LOWESS technique. A-I is sequential labelling of the nine selected hospitals in this figure while H e.g., H1 corresponds to all 22 hospitals in supplementary figure 1. CIN, Clinical Information Network; First Line, penicillin and gentamicin. Third Gen, third generation; Others, other antibiotics.

Considerable variation in average monthly admission antibiotic prescriptions was however observed *between hospitals* and *within hospitals over time* for all the antibiotic categories as shown in Fig. 4 (9 hospitals) and Supplementary Figure S1 (all 22 hospitals. The 9 hospitals in Fig. 4 were purposively selected to illustrate the extremes of variation. The prescription of third generation cephalosporins is illustrative with hospital specific use ranging from of 0–30% at the beginning of the study period (Supplementary Figure S1) while a peak of 100% was observed in two facilities (H1 and H6, Fig. 4). Even in the tertiary facility (H = 7) first line prescriptions generally accounted for greater than 80% monthly antibiotic use at admission. At two time points however, this dropped to less than 60% and 50% with a commensurate greater 30% and 40% prescription of the “others” category. Peak third generation cephalosporin prescription (≈10%) corresponded to these two periods.

**Fig. 5:**
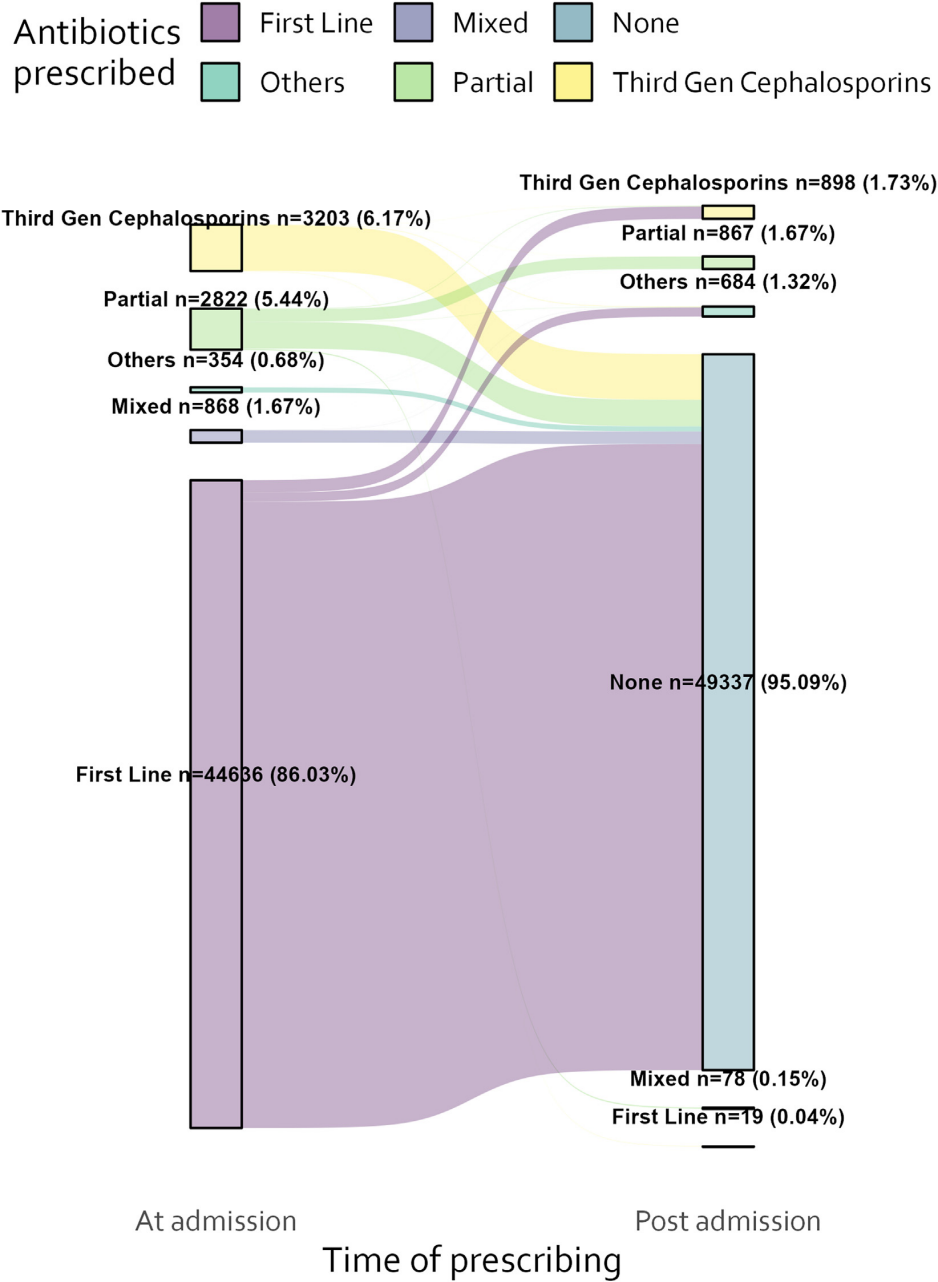
**Exploring post admission antibiotic switching amongst patients with an antibiotic prescription at admission**. First line: Gentamicin and Penicillin prescribed together without other antibiotics; Partial: Gentamicin OR Penicillin only prescribed (but not both); Mixed: Gentamicin OR Penicillin prescribed together with other antibiotics; Others: Any other antibiotic.

### Factors associated with non-first line antibiotic prescriptions at admission

A multivariable random effects logistic regression model that included 8 covariates was used to examine the association with *probability of non-first line antibiotic prescription* at admission. Four of these -SENSS score, Apgar at 5 min < 7, being outborn (this includes babies admitted following delivery at another facility or a non-facility delivery e.g. home birth) and temperature at admission-were associated with a greater adjusted odds of non-prescription of first line antibiotics (Table 2). The largest magnitude of effect was observed for outborn babies compared to the inborn where the odds of non-first line prescription was more than twice (OR 2.27, 95% CI 2.12–2.43).

**Table 2:** Factors associated with none-first line antibiotic prescription at admission.

Characteristic	aOR	95% CI	p-value
Time^a^, ^b^	1.24	0.90, 1.71	0.179
CIN history^a^, ^c^	0.93	0.39, 2.26	0.880
NEST360^d^	0.82	0.34, 1.98	0.664
SENSS score^e^	1.03	1.00, 1.05	0.022
Apgar at 5 min < 7^f^	1.54	1.44, 1.65	<0.001
SPO_2_ < 90%^g^	1.08	1.00, 1.17	0.062
Outborn^h^	2.27	2.12, 2.43	<0.001
Temperature^i^	1.11	1.08, 1.14	<0.001

a Variable is log-transformed.

b Effect of time over the study period.

c Duration of CIN (Clinical Information Network) membership in months before the commencement of this study (Supplementary Table S1).

d Participation in the NEST360 (Newborn Essential Solutions and Technologies) programme.

e SENSS (Score of Essential Newborn Symptoms and Signs) score provided as a mortality risk probability (derived from sex, birthweight, presence or absence of difficulty feeding, convulsions, indrawing, central cyanosis and floppy/inability to suck).

f Apgar score measured at 5 min after birth.

g SPO_2_: Oxygen saturation measured by pulse oximetry.

h Born either at another facility or not born in a facility.

i Temperature at admission, variable centered at the mean.

### Estimated antibiotic consumption at admission

Antibiotic consumption across the twenty-two facilities was estimated based on prescription rather than documented administration. The median length of therapy for individual patients was 0.75 per day spent in hospital (750/1000 patient days). This means that by this metric patients spent 75% of their time in hospital on *any* antibiotic prescribed at admission (Table 3). This was less at hospital level at 42% (418/1000 patient days).

**Table 3:** Antibiotic use metrics based on prescription at admission at patient level.

Metric	Statistic (median, IQR)	
	Per 1000 average days of stay (patient level)	Per 1000 patient day (center level)
Prescribed length of therapy (LOT)^a^	750 (330–1000)	418 (389–500)
Prescribed days of therapy (DOT)^b^	1200 (600–2000)	744 (691–869)

IQR: Interquartile range.

a LOT number of days with any antibiotic divided by length of stay or patient days.

b DOT number of days with ≥1 antibiotic divided by length of stay or patient days (each drug counted separately).

Considering multiple prescriptions with days of therapy, at patient level this was 1200 and at center level 744. This means on average there was a prescription of 1.2 days of antibiotics for each patient day (length of stay). Given that the bulk of prescriptions were first line it follows that the DOT (Penicillin and Gentamicin counted separately) is roughly twice the LOT (Penicillin and Gentamicin counted together), and the former reflects the current Ministry of Health policy on first line antibiotics.

### Exploring postadmission antibiotic prescription and switching

Post admission antibiotic data were not systematically collected across all hospitals and throughout the study period. All hospitals had at least one patient with a post admission antibiotic prescription with a range of 2–440 prescriptions (Supplementary Table S2). These data are therefore not representative but a descriptive exploration. Post admission antibiotics prescriptions were observed in two populations (1) those who had a prescription at admission (Fig. 1, Population C, n = 2546) and those who didn't (Fig. 1, Population D (n = 9647) for a total of 12,193 post admission prescriptions. For those with admission antibiotic prescriptions, the bulk of the post admission prescriptions (44%, 1131/2546) were for day 7–28 (Supplementary Figure S2) and so were in neonates with extended inpatient stays given the median length of stay of 5 days (IQR 2–9 days, Table 2).

Fig. 5 Explores switching amongst those who had a prescription at admission (Fig. 1 population A (n = 51,833) and population C (n = 2546). The majority of those who had first line at admission didn't have a further prescription post admission (95.09%, 49,337/51,883). Amongst those who did, the most common switch was to third generation cephalosporins (Fig. 5). No patients who had third generation cephalosporins at admission had a further prescription post admission.

## Discussion

This study included 82,834 neonates from 22 Kenyan neonatal units admitted between 1st September 2020 and 31st October 2023. Almost two thirds of the neonates (62.63%, 51,883/82,834) had an antibiotic prescription at admission and 14.82% (12,193/82,834) got antibiotics post admission. Out of these 12,193, 9647/12,193 (79.12%) were getting antibiotic treatment for the first time during their inpatient stay, while 2546/12,193 (20.88%) were getting antibiotic treatment as a switch form the treatment given at admission. Amongst those with antibiotics at admission, the most common diagnoses were low birth weight, respiratory distress syndrome, birth asphyxia and neonatal sepsis.

The most prescribed antibiotics at admission was the combination of crystalline penicillin and gentamicin (first line as per the national guidelines^18^) accounting for an average of 86% of antibiotic prescriptions at admission. This is consistent with a previous point prevalence survey which in addition demonstrated wide variation in antibiotic prescriptions in adult (medical and surgical) and paediatric surgical wards.^22^ This likely reflects the dissemination of paediatric and neonatal medical guidelines since 2016 and for the facilities included in this study, the linked networking within the CIN.^15^ This first line antibiotic use is similar to African sites in the Burden of Antibiotic Resistance in Neonates from Developing Societies (BARNARDS) study where ampicillin-gentamicin was the most frequent combination.^23^ However, the 12 sites included in BARNARDS are largely urban tertiary centres where the other 3 most common combinations were ceftazidime–amikacin, piperacillin–tazobactam–amikacin and amoxicillin clavulanate–amikacin which are not necessarily generalisable to non-tertiary hospitals.^23^

There was little variation in this using aggregate data over the study period but significant within hospital variation was observed with peak third generation cephalosporin (ceftriaxone and ceftazidime) at 100% in two facilities. The odds of non-prescription of first line antibiotics was increased with severity of illness (SENNS score), Apgar at 5 min < 7, SPO_2_ < 90%, outborn and higher temperature with the largest effect for outborn versus inborn (OR 2.27, 95% CI 2.12–2.43) shown in These effects may however be small relative to the effect of variation at hospital level as illustrated in Fig. 4. Penicillin and gentamicin dominated antibiotic consumption resulting in days of therapy (DOT) approximately twice the length of therapy (LOT). Finally, the exploratory post admission antibiotic data suggests that there was a modest number of postadmission antibiotic switching and amongst these, third generation cephalosporins were the most common (Fig. 3).

The high antibiotic prescription at admission (62.63%) is comparable to the 70% prevalence at admission in four African countries reported by Murless-Collins et al. that included data from Kenyan neonatal units.^5^ Other data on prevalence of antibiotic prescriptions in were not restricted to admission prescriptions but also included post admission antibiotics. Our current estimate is however still higher than 51.6% in India,^24^ 44.8% from recent USA data^10^ and 41.4% in Indonesia^25^ but lower than 88.4% in China.^12^ This may be related to differences in severity of illness at admission (case mix variation) and the limited diagnostic microbiology capacity.

First line (penicillin and gentamicin) were the most prescribed antibiotics on average throughout the study period similar to findings of a point prevalence study by Hsia et al. for African neonates.^26^ Penicillin and gentamicin are both classified as ‘access’ antibiotics in the World Health Organisation AWaRe (access, watch, reserve) classification for appropriate use and risk of development of resistance.^7^ However of concern is between and within hospital variation especially in the use of third generation cephalosporins that are within the ‘watch’ category. Based on discussions with study hospital teams during data quality assurance exercises, the instances of peak 100% cephalosporin use in some hospitals was due to stockouts of first line antibiotics. The frequent use of these class of cephalosporins was also noted by Russel et al.^27^ In their study of 3204 infants aged <60 days, 13.8% were started on either cefotaxime or ceftriaxone for clinical sepsis. The usage of cephalosporins has long been recognised as a driver of development of drug resistant pathogens and in neonates has been associated with other adverse outcomes including invasive fungal infections.^6^ Perhaps reassuringly we saw very limited use of ‘reserve’ antibiotics (tigecycline).

In addition to the strong influence of specific hospital context prescription of non-first line antibiotics at admission was associated with clinical characteristics suggesting increased severity of illness influenced decision making. Neonatal sepsis in many LMIC settings with limited access to diagnostic microbiology is a clinically heterogenous condition with a broad definition in Kenyan and WHO clinical practice guidelines.^18^ Antibiotic prescription is a complex phenomenon influenced by knowledge, attitude and practices spanning the patient's condition, the environmental context and availability of resources.^28^ Efforts to further rationalise admission antibiotic use in hospitals like those studied may require development and pragmatic testing of more specific, risk based clinical guidance ideally linked to improved access to diagnostics.

Antimicrobial resistance (AMR) is especially important in low resource settings which face the largest burden and the attendant morbidity and mortality.^29^ Determining the effectiveness of strategies that aim to limit AMR will require that we can measure antibiotic consumption in facilities as part of antimicrobial stewardship programmes.^14^ The estimates of length of therapy (LOT) and days of therapy (DOT) provide measures of antibiotic consumption appropriate for neonates and allow for comparison across different settings. The DOT in our study was 1200 (600–2000) per 1000 days length of stay and 744 (691–869) per 1000 patient days and is largely driven by adherence to the guideline recommendation for combination therapy. These results are similar to the median reported by Cartledge et al. from Rwanda at 800 per 1000 days.^13^ The DOT per 1000 patient days is however approximately three times the 274 per 1000 in USA and almost twice the 441 per 1000 in China.^10^^,^^12^ Our research indicates antibiotic use can be assessed at scale over prolonged periods with improved health information systems. Where comprehensive, routine monitoring is not possible alternative metrics include antibiotic use measured by point prevalence surveys or through specific antibiotic audits. Data generated should be given as feedback to prescribers.^14^ The regular feedback on adherence to guidelines within the Kenyan Clinical Information Network may have contributed to relatively high levels of first-line antibiotic recommendations across the 3 years period studied.^15^

The first limitation of this study is only admission antibiotic prescription data was available for all study participants. Postadmission prescriptions were not systematically collected but were from instances where data clerks abstracted all antibiotic prescriptions over the course of admission rather than just those on admission that were required by the standard operating procedure. These data however provided a glimpse of antibiotic switching post admission. Secondly the LOT and DOT were computed based on days prescribed rather than actual days administered. This is a feature of secondary analysis of a data set not designed specifically to measure antibiotic consumption. However, we suggest prescription data is a reasonable proxy for individual patient consumption especially as actual medicines administration data (consumption) is hard to collect at large scale in LMIC hospitals.

In addition, there was lower case facility, lower severity of illness (SENSS score) and shorter length of stay in those with missing sheets. This suggests this population was less severely ill and their exclusion may have resulted in an overestimate of antibiotic use. For the all variables except treatment sheet variables, the probability for missing values in one variable is unrelated to the probability of missing data in another variable (missing at random, MAR); The reason why the missingness of the variables in the treatment sheets is not considered MAR, but rather MNAR (missing not at random), is because the data missing are systematically related to unobserved events/factors that we do not measure (e.g. stock-out of treatment sheets, treatment sheets removed from the patient record and stored separately etc.). A key strength of these data however is the prospective nature allowing description of trends over time which is not possible with point prevalence surveys.

Our results show that there is high antibiotic consumption with most of it first line as per national standard treatment guidelines (benzyl penicillin and gentamicin). However, there is also significant use of non-first line antibiotics especially third generation cephalosporins that may be in part driven by clinicians assessment of severity of illness in a setting with minimal capacity for microbiology diagnostic testing. To reduce consumption, antimicrobial stewardship programmes need to be established and tested in keeping with the National Action Plan on antimicrobial resistance, and we have demonstrated the feasibility and magnitude of potential antibiotic consumption metrics from observational data that aligns with the National Antimicrobial Stewardship Guidelines recommendation on monitoring of antibiotic use to assess such programmes.

## Contributors

JA and ME conceptualised the study. MO oversaw the data collection and operation of the network supported by JA and ME. TT led the analyses with support from JA and ME. JA and TT had full access to all data in the study and take responsibility for verification of the data, and the accuracy of the data analysis. JA drafted the initial manuscript. Feedback on subsequent manuscript drafts were provided by all authors. All authors read and approved the final manuscript.

## Data sharing statement

Data are available in Harvard Dataverse. Applications for access can be made through the KWTRP Data Governance Committee with details available on www.kemri-wellcome.org, or email to dgc@kemri-wellcome.org. The datasets generated and/or analysed during the current study are not publicly available due to the primary data being owned by the hospitals and their counties with the Ministry of Health; The research staff do not have permission to share the data without further written approval from both the KEMRI-Wellcome Trust Data Governance Committee and the Facility, County or Ministry of Health as appropriate to the data request. Requests for access to primary data from quantitative research by people other than the investigators will be submitted to the KEMRI-Wellcome Trust Research Programme data governance committee as a first step through dgc@kemri-wellcome.org who will advise on the need for additional ethical review by the KEMRI Research Ethics Committee.

## Declaration of interests

The authors declare that they have no competing interests.

## Appendix A Supplementary data

Supplementary Figures and Tables

CIN Author
