# Supplementary material for: Situational analysis of antibiotic prescriptions in Kenyan neonatal units for antimicrobial stewardship: a retrospective longitudinal study
Source: eClinicalMedicine. 2025 Mar 26;82:103156. doi: 10.1016/j.eclinm.2025.103156 (PMC11985152; doi:10.1016/j.eclinm.2025.103156)
Supplement: Supplementary Figures and Tables [file mmc1.docx]

**Situational analysis of antibiotic prescriptions in Kenyan neonatal units for antimicrobial stewardship: A retrospective longitudinal study**

Jalemba Aluvaala DPhil ^a, b, d^, Timothy Tuti DPhil ^a^, Muthoni Ogola PhD ^a^, Cherry Lim DPhil ^c^, Sean Cavany PhD ^c^, Mike English MD ^a, c^; The Clinical Information Network Author Group

**Supplementary Appendix**

**Table 1: Summary of antibiotic use in neonatal units**

| **Authors** | **Year** | **Country** | **Site** | **Study population** | **Antibiotic use metric** | **Result** |
| --- | --- | --- | --- | --- | --- | --- |
| Murless‑Collins et al^5^ | 2023 | Kenya, Malawi, Tanzania, Nigeria | 61 hospitals | 144,146 newborns  (2019 to 2022) | Prevalence | 70% |
| Flannery et al^10^ | 2023 | USA | 735 NICUs* | 1,395,791  (2009 to 2021) | Prevalence  DOT per 1000 patient days | 44·8%  274 per 1000 |
| Salsabila et al^14^ | 2022 | Indonesia | 3 neonatal units | 2565  day 0 to 3 of life | Prevalence | 41·4% (1039/2565) |
| Spencer et al^8^ | 2022 | USA | 51 NICUs* | 2813 infants  (2017) | Point prevalence | 23% received at least one antibiotic |
| Boverman et al^11^ | 2022 | USA | 6 NICUs* | All admissions at birth from 2010 to 2017 (n=15,015) | Number of days of antibiotics per 100 patient-days | Decline from 15·7 -16·6 to 10·1 – 10·8% |
| Jiang et al^12^ | 2021 | China | 25 NICUs* | All admissions <34 weeks 2015 to 2018 (n=24597) | Prevalence  Days per 1000 patient days | 88·4%  Median 441 per 1000 |
| Cartledge et al^13^ | 2020 | Rwanda | 3 neonatal units | Inborn admitted neonates given a course of antibiotics.  (n=126) | Days per length of stay | Median use ratio 0·8 |
| Gandra et al^15^ | 2018 | India | 8 NICUs* | All admissions 2016 to 2017  (n=403) | Four-point prevalence surveys | 51·6% prescribed one or more antimicrobials |
| Carr et al^16^ | 2016 | Australia | 1 NICU* | All admissions 1990 to 2014 (n=20,962) | Proportion that received, mean duration | Decline in % by 0·27% per year |

***NICU: Neonatal Intensive Care Unit

**Table 2: Characteristics of neonatal units included in the study**

| **Hospital Code** | **CIN-N Join Date** | **Months in CIN pre-study** | **Total Patients**  **in study** | **Median Monthly Patients**  **(IQR)_** | **Patients with post admission**  **Antibiotics** |
| --- | --- | --- | --- | --- | --- |
| H1 | Nov-18 | 22 | 6171 | 49 (31-60) | 11 |
| H2 | Feb-18 | 31 | 4125 | 24 (14-32) | 30 |
| H3 | Mar-18 | 30 | 7453 | 33 (20-43) | 2 |
| H4 | Jun-19 | 15 | 1469 | 17 (13-24) | 22 |
| H5 | Nov-18 | 22 | 5722 | 72 (62-83) | 9 |
| H6 | Apr-18 | 29 | 8278 | 28 (18-38) | 81 |
| H7 | Dec-19 | 9 | 10019 | 142 (118-173) | 247 |
| H8 | Mar-18 | 30 | 6255 | 25 (19-34) | 32 |
| H9 | Oct-18 | 23 | 12197 | 88 (75-101) | 89 |
| H10 | Mar-18 | 30 | 2920 | 36 (27-46) | 85 |
| H11 | Apr-18 | 29 | 6846 | 34 (25-47) | 81 |
| H12 | Mar-18 | 30 | 12831 | 57 (48-66) | 271 |
| H13 | Jun-17 | 39 | 12908 | 121 (85-137) | 440 |
| H14 | Mar-18 | 30 | 6978 | 69 (53-97) | 77 |
| H15 | Nov-19 | 10 | 4329 | 49 (41-57) | 414 |
| H16 | Sep-19 | 12 | 6947 | 116 (103-131) | 289 |
| H17 | Sep-19 | 12 | 9516 | 138 (67-150) | 61 |
| H18 | Apr-18 | 29 | 5797 | 18 (15-20) | 22 |
| H19 | Apr-14 | 77 | 38637 | 193 (173-213) | 138 |
| H20 | Oct-18 | 23 | 11185 | 57 (53-67) | 9 |
| H21 | Mar-18 | 30 | 2493 | 28 (24-36) | 81 |
| H22 | Jul-17 | 38 | 1676 | 15 (12-18) | 55 |

**Table 3: Admission diagnoses in neonates with antibiotic prescriptions at admission**

| **Diagnosis** | **Counts** | **%** |
| --- | --- | --- |
| Others | 18154 | 18.6 |
| Respiratory Distress Syndrome | 17132 | 17.56 |
| Low Birth Weight | 16529 | 16.94 |
| Neonatal Sepsis | 15500 | 15.88 |
| Birth Asphyxia | 14646 | 15.01 |
| Meconium Aspiration | 6893 | 7.06 |
| Neonatal Jaundice | 3592 | 3.68 |
| Tachyponea | 1885 | 1.93 |
| Dehydration | 1014 | 1.04 |
| Congenital Anomalies | 633 | 0.65 |
| Macrosomia | 541 | 0.55 |
| Meningitis | 449 | 0.46 |
| Twin | 364 | 0.37 |
| Well baby admitted for accommodation | 245 | 0.25 |

**Table 4: Summary of all antibiotics prescribed**

|  | **Class** | **Generation** | **Tertiary Hospital** | | | | **Other Hospitals** | | | |
| --- | --- | --- | --- | --- | --- | --- | --- | --- | --- | --- |
|  |  |  | **n** | % | **Class (n)** | **%** | **n** | **%** | **Class (n)** | **%** |
| Amikacin | Aminoglycoside |  | 313 | 2.85 | 4893 | **44.55%** | 1099 | 116.00% | 42583 | **45.10%** |
| Gentamicin | Aminoglycoside |  | 4580 | 41.7 |  |  | 41484 | 4394.00% |  |  |
| Meropenem | Carbapenem |  | 101 | 0.92 | 101 | **0.92%** | 149 | 16.00% | 149 | **0.16%** |
| Cefazolin | Cephalosporin | 1 | 2 | 0.02 | 2 | **0.02%** | 0 | 0.00% | 0 | **0.00%** |
| Cefepime | Cephalosporin | 4 | 24 | 0.22 | 24 | **0.22%** | 4 | 0.00% | 4 | **0.00%** |
| Cefixime | Cephalosporin | 3 | 1 | 0.01 | 723 | **6.58%** | 10 | 1.00% | 5830 | **6.17%** |
| Cefotaxime | Cephalosporin | 3 | 4 | 0.04 |  |  | 14 | 1.00% |  |  |
| Ceftazidime | Cephalosporin | 3 | 689 | 6.27 |  |  | 4854 | 514.00% |  |  |
| Ceftriaxone | Cephalosporin | 3 | 29 | 0.26 |  |  | 952 | 101.00% |  |  |
| Cefuroxime | Cephalosporin | 2 | 2 | 0.02 | 2 | **0.02%** | 142 | 15.00% | 278 | **0.29%** |
| Cefuroxime-Sulbactam | Cephalosporin | 2 | NA | NA |  |  | 136 | 14.00% | 136 | **0.14%** |
| Vancomycin | Glycopeptides |  | 2 | 0.02 | 2 | **0.02%** | 21 | 2.00% | 21 | **0.02%** |
| Tigecycline | Glycylcycline |  | 1 | 0.01 | 1 | **0.01%** | 0 | 0.00% | 0 | **0.00%** |
| Clindamycin | Lincosamide |  | 1 | 0.01 | 1 | **0.01%** | 1 | 0.00% | 1 | **0.00%** |
| Metronidazole | Nitroimidazole |  | 37 | 0.34 | 37 | **0.34%** | 1668 | 177.00% | 1668 | **1.77%** |
| Amoxicillin | Penicillin | **Other** | 27 | 0.25 | 207 | **1.88%** | 50 | 5.00% | 784.0003 | **0.83%** |
| Amoxicillin-Clavulanate | Penicillin | Other | 32 | 0.29 |  |  | 54 | 6.00% |  |  |
| Amoxicillin-Flucloxacillin | Penicillin | Other | 1 | 0.01 |  |  | 0 | 0.00% |  |  |
| Amoxycillin | Penicillin | Other | 1 | 0.01 |  |  | 0 | 0.00% |  |  |
| Ampicillin | Penicillin | 1 | 10 | 0.09 | 4983 | **45.37%** | 39 | 4.00% | 43060 | **45.61%** |
| Benzathine Penicillin | Penicillin | 1 | 1 | 0.01 |  |  | 2 | 0.00% |  |  |
| Benzyl Penicillin | Penicillin | 1 | 1 | 0.01 |  |  | 48 | 5.00% |  |  |
| Cloxacillin | Penicillin | Other | 1 | 0.01 |  |  | 6 | 1.00% |  |  |
| Flucloxacillin | Penicillin | Other | 139 | 1.27 |  |  | 664 | 70.00% |  |  |
| Penicillin | Penicillin | 1 | 4971 | 45.26 |  |  | 42971 | 4551.00% |  |  |
| Piperacillin-Tazobactam | Penicillin | Other | 6 | 0.05 |  |  | 6 | 1.00% |  |  |
| Ampicilin-Cloxacillin | Penicillin | Other | 0 | NA |  |  | 1 | 0.00% |  |  |
| Floxacillin | Penicillin | Other | 0 | NA |  |  | 3 | 0.00% |  |  |
| Ciprofloxacin | Quinolone |  | 3 | 0.03 | 3 | **0.03%** | 23 | 2.00% | 23 | **0.02%** |
| Cotrimoxazole | Sulfonamide |  | 4 | 0.04 | 4 | **0.04%** | 13 | 1.00% | 13 | **0.01%** |
| **Total** |  |  | **10983** |  |  |  | **94414** |  |  |  |

**Table 5: The most prescribed antibiotics at admission**

| **Prescription** | **Count** | **Proportion** |
| --- | --- | --- |
| Amikacin | 1220 | 1.2 |
| Ampicillin | 42 | 0.04 |
| Ceftazidime | 5155 | 5.09 |
| Ceftriaxone | 879 | 0.87 |
| Gentamicin | 46008 | 45.44 |
| Penicillin | 47942 | 47.35 |


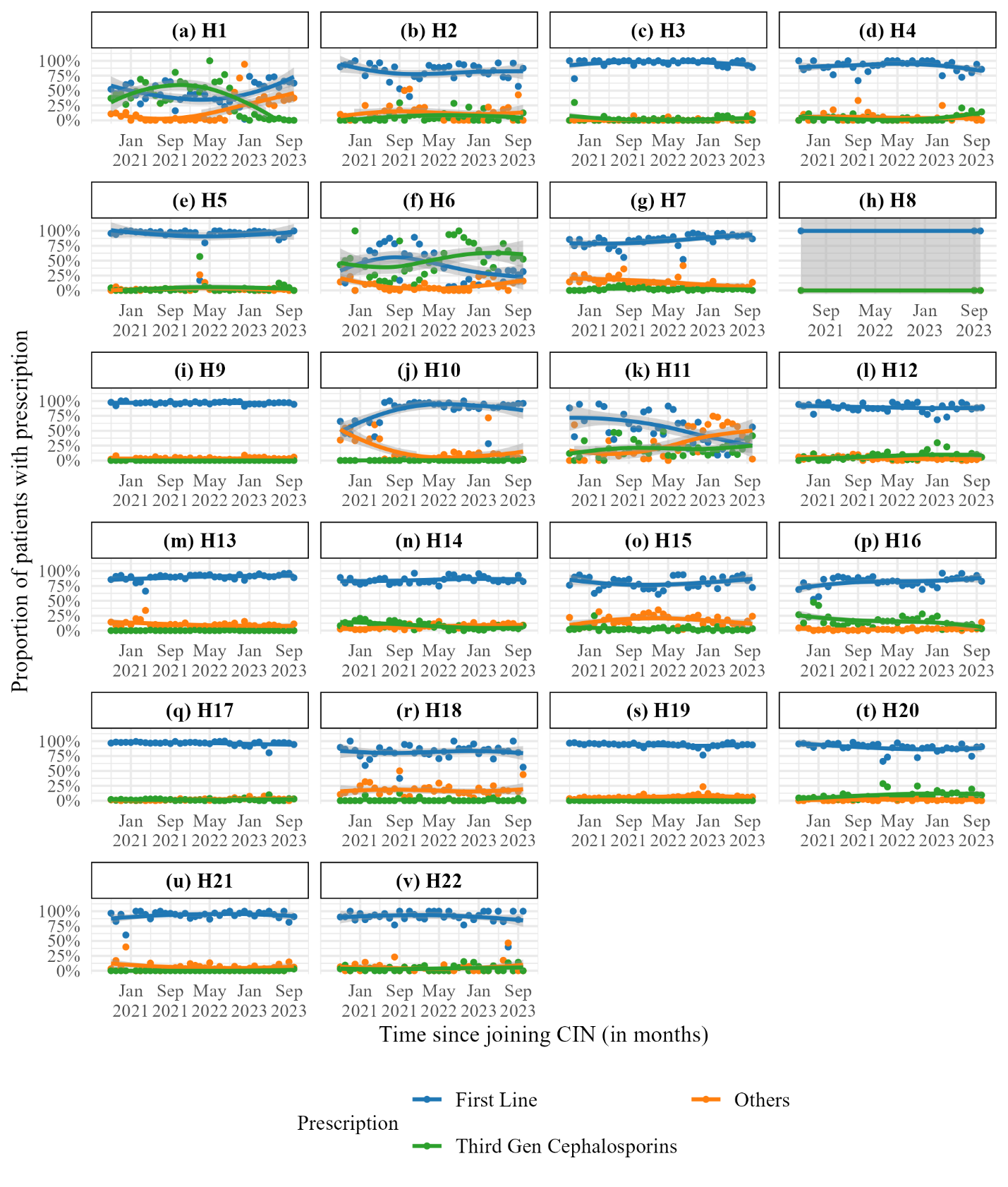


**Figure 1:** **Variation in antibiotic prescription patterns at admission in the twenty-two hospitals over time**


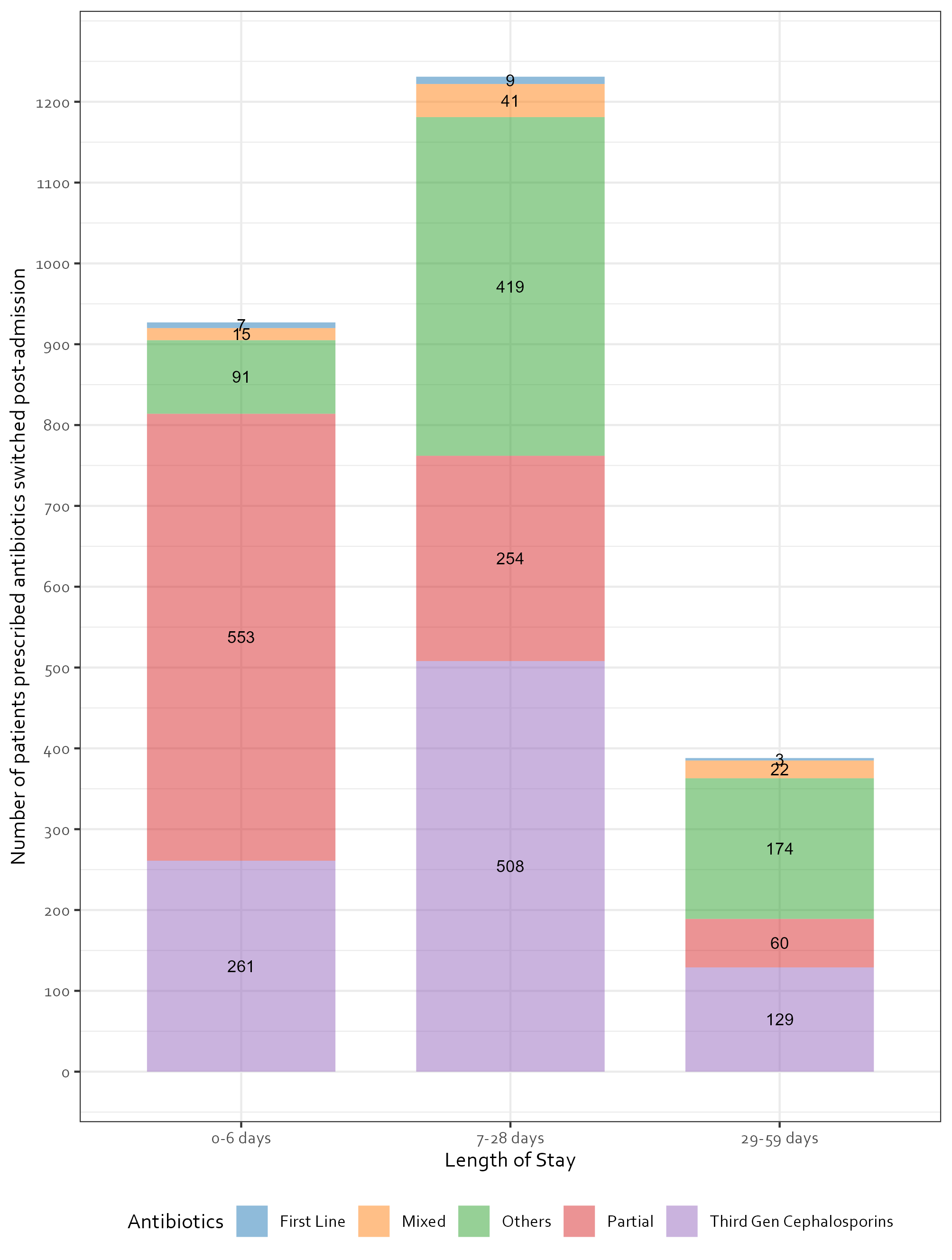


**Figure 2: Timing of new antibiotic prescriptions postadmission amongst neonates with an admission prescription**


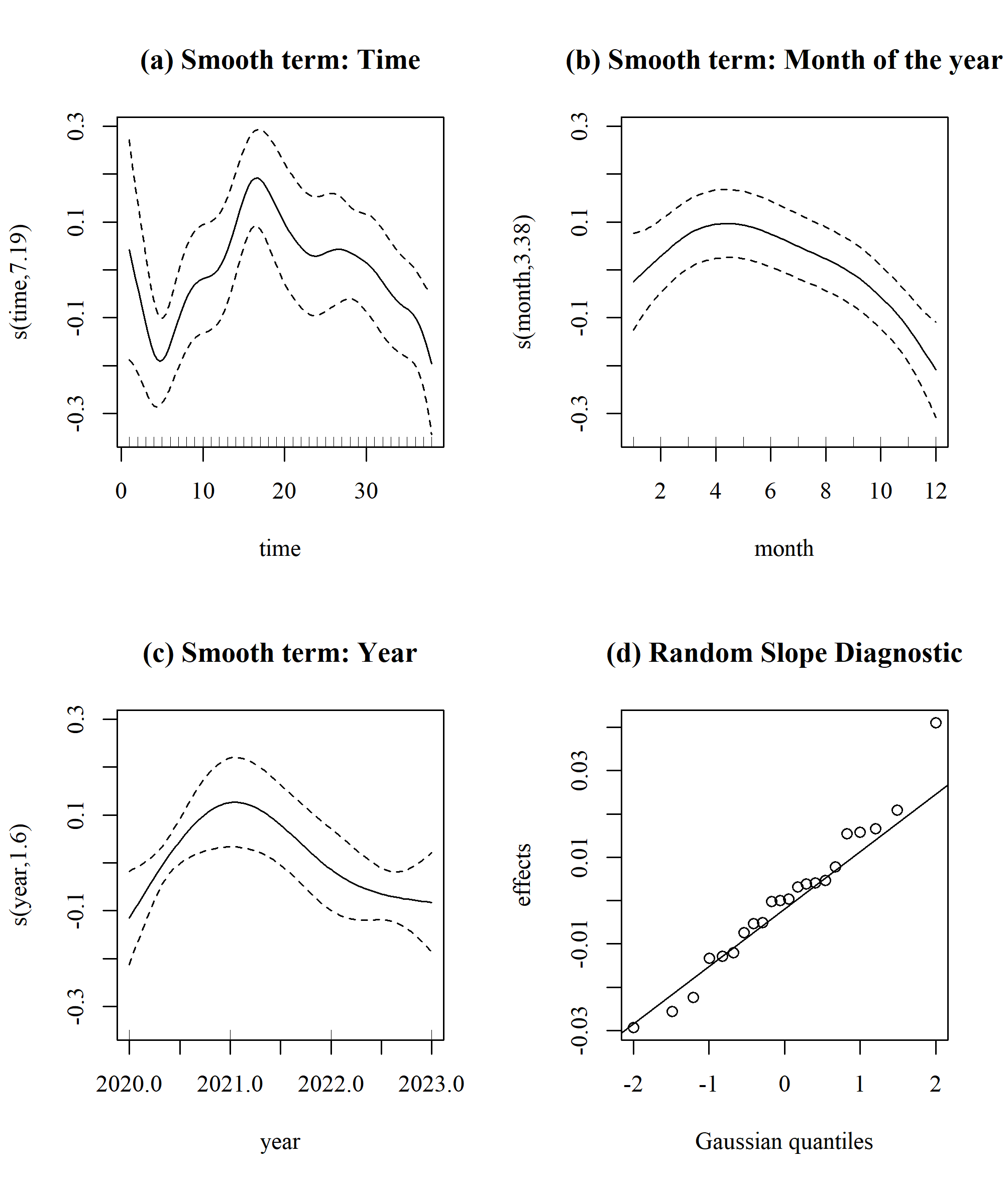


**Figure 3: Trend analysis of prescription of first line antibiotics**
