# Supplementary material for: Situational analysis of antibiotic prescriptions in Kenyan neonatal units for antimicrobial stewardship: a retrospective longitudinal study
Source: eClinicalMedicine. 2025 Mar 26;82:103156. doi: 10.1016/j.eclinm.2025.103156 (PMC11985152; doi:10.1016/j.eclinm.2025.103156)
Supplement: CIN Author [file mmc2.docx]

| **First Names** | **Surnames** |
| --- | --- |
| Dolphine | Mochache |
| Florence | Murila |
| Wairimu | Kimani |
| Duncan | Chabi |
| Lilian | Naibei |
| Juma | Vitalis |
| Amilia | Ngoda |
| Geoffrey Habil | Shikanda |
| Nyumbile | Bonface |
| Roselyn | Malangachi |
| Ijusa | Midecha |
| Eileen | Muhavi |
| Samuel | Soita |
| Christine | Manyasi |
| Catherine | Mutinda |
| Zanuba | Mohammed |
| Rukia | Aden |
| Rebecca | Toroitich |
| Joyce | Mbogho |
| Dion | Nzoki |
| Joseph | Ng’ang’a |
| Celia | Kariuki |
| Cecilia | Mutiso |
| Elizabeth | Jowi |
| Josephine | Aritho |
| Beatrice | Njambi |
| Benjamin | Wambua |
| Esther | Mwangi |
| Charles | Nzioki |
| Penina | Musyoka |
| Zainabu | Kioni |
| Miriam | Munyalo |
| Esther | Muthiani |
| Carol | Ntii |
| Esther | Njeri |
| Agnes | Mithamo |
| Lucy | Kinyua |
| Faith | Kimotho |
| Magdalene | Kuria |
| Alice | Oguda |
| Mary | Akoth |
| Christine | Marete |
| Loise | Mwangi |
| Mukami | Becky |
| Penina | Mwangi |
| Nancy | Mburu |
| Juliet | Gachoki |
| Rachel | Inginia |
| Paul | Njanwe |
| Mwende | Mutunga |
| Celestine | Muteshi |
| Ann | Chebet |
| Emma | Namulala |
| Yuvane | Maiyo |
| Salome | Muyale |
| Susan | Wanjala |
| Grace | Ochieng |
| Catherine | Murianki |
| Lydia | Thuranira |
| Virginiah | Njoki |
| Margaret | Waweru |
| Faith | Mumo |
| Felistus | Makokha |
| Maureen | Natembea |
| Francis | Soita |
| Josephine | Ojigo |
| Maureen | Muchela |
| Don | Ogollah |
| Joyce | Oketch |
| Assenath | Okeyo |
| Rashid | Musa |
| Beth | Maina |
| Maureen | Muriithi |
| Bashir | Denkwo |
| Orina | Nyakina |
| Faith | Njeru |
| Judith | Onsongo |
| Lucy | Lyanda |
| Mwangi | Wagura |
| Catherine | Githaiga |
| Consolata | Kinyua |
| Linda | Ombito |
| Alice | Nkirote |
| Elizabeth | Kibaru |
| Caroline | Limo |
| Benjamin | Tanui |
| Patricia | Muiruri |
| Bernadette | Lusweti |
| Patrick | Mburugu |
| Sylvia | Mwathi |
| Maureen | Njoroge |
| Marion | Kiguoya |
| Jane | Ndege |
| Peter | Muigai |
